# Immunopharmacology and Quantitative Analysis of Tyrosine Kinase Signaling

**DOI:** 10.1002/cpim.104

**Published:** 2020-09-15

**Authors:** Ben F. Brian, Candace R. Guerrero, Tanya S. Freedman

**Affiliations:** ^1^ Department of Pharmacology University of Minnesota Minneapolis Minnesota; ^2^ College of Biological Sciences Center for Mass Spectrometry and Proteomics University of Minnesota Minneapolis Minnesota; ^3^ Center for Immunology, Masonic Cancer Center, Center for Autoimmune Diseases Research University of Minnesota Minneapolis Minnesota

**Keywords:** immunopharmacology, phosphorylation, protein tyrosine kinase, quantitative immunoblot, targeted mass spectrometry, Western blot

## Abstract

In this article we describe the use of pharmacological and genetic tools coupled with immunoblotting (Western blotting) and targeted mass spectrometry to quantify immune signaling and cell activation mediated by tyrosine kinases. Transfer of the ATP γ phosphate to a protein tyrosine residue activates signaling cascades regulating the differentiation, survival, and effector functions of all cells, with unique roles in immune antigen receptor, polarization, and other signaling pathways. Defining the substrates and scaffolding interactions of tyrosine kinases is critical for revealing and therapeutically manipulating mechanisms of immune regulation. Quantitative analysis of the amplitude and kinetics of these effects is becoming ever more accessible experimentally and increasingly important for predicting complex downstream effects of therapeutics and for building computational models. Secondarily, quantitative analysis is increasingly expected by reviewers and journal editors, and statistical analysis of biological replicates can bolster claims of experimental rigor and reproducibility. Here we outline methods for perturbing tyrosine kinase activity in cells and quantifying protein phosphorylation in lysates and immunoprecipitates. The immunoblotting techniques are a guide to probing the dynamics of protein abundance, protein–protein interactions, and changes in post‐translational modification. Immunoprecipitated protein complexes can also be subjected to targeted mass spectrometry to probe novel sites of modification and multiply modified or understudied proteins that cannot be resolved by immunoblotting. Together, these protocols form a framework for identifying the unique contributions of tyrosine kinases to cell activation and elucidating the mechanisms governing immune cell regulation in health and disease. © 2020 The Authors.

**Basic Protocol 1**: Quantifying protein phosphorylation via immunoblotting and near‐infrared imaging

**Alternate Protocol**: Visualizing immunoblots using chemiluminescence

**Basic Protocol 2**: Enriching target proteins and isolation of protein complexes by immunoprecipitation

**Support Protocol**: Covalent conjugation of antibodies to functionalized beads

**Basic Protocol 3**: Quantifying proteins and post‐translational modifications by targeted mass spectrometry

## INTRODUCTION

Tyrosine kinases are critical mediators of immune cell activation and regulation (Hwang, Byeon, Kim, & Park, 2020; Lowell, 2011). The transfer of the ATP γ phosphate to a protein tyrosine residue initiates signaling cascades that alter cell survival, proliferation, and effector functions. The steric and electrostatic effects of tyrosine phosphorylation can induce conformational changes in proteins that expose docking sites, block autoinhibitory interactions, or deprotect motifs for trafficking, degradation, or further post‐translational modification. Phosphotyrosine‐containing peptides also serve as direct SH2 and PTB binding sites, nucleating higher‐order signaling complexes that tune signal strength and kinetics and may even alter the phase properties of signaling complexes (Case, Ditlev, & Rosen, 2019; Oh et al., 2012). The actions of tyrosine kinases initiate an array of immune cell functions, including pathogen detection and killing, phagocytosis, clonal expansion, and migration to sites of infection or damage.

Accordingly, dysregulation of tyrosine kinase signaling pathways is associated with many diseases, including autoimmune and inflammatory disease and cancer. Analysis of activated signaling pathways, therefore, is critical for understanding how immune cells participate in health and disease.

In this article we highlight genetic and chemical tools—including competitive inhibitors, designer kinase–inhibitor pairs, small interfering RNA (siRNA), and CRISPR/Cas9 gene editing—for dissecting tyrosine kinase signaling in immune cells. We present protocols for quantitative evaluation of signaling kinetics, amplitude, and binding interactions and for identifying sites of post‐translational modification. Our protocols feature adherent bone marrow–derived macrophages (BMDMs), but we describe adaptations for use with lymphocytes and other cells in suspension. Basic Protocol 1 describes a method for quantitative immunoblotting. Basic Protocol 2 describes a method for (co‐)immunoprecipitation of proteins from cell lysates, which can be used in conjunction with immunoblotting or quantitative, targeted mass spectrometry described in Basic Protocol 3. These cell perturbation and protein enrichment strategies can also be used as precursors to flow cytometry or proteomic methods (see Current Protocols articles: Breitkopf & Asara, 2012; Schulz, Danna, Krutzik, & Nolan, 2012).

## Basic Protocol 1 QUANTIFYING PROTEIN PHOSPHORYLATION VIA IMMUNOBLOTTING AND NEAR‐INFRARED IMAGING

The procedure for immunoblotting (Western blotting) was developed in the early 1980s. Subsequent advances in monoclonal antibody production, secondary antibody fluorophore conjugation, transfer methods, visualization strategies, and methods for quantification have made immunoblotting a workhorse method for quantifying biochemical changes in cells (Janes, 2015). In this protocol denatured cell lysates are resolved by size via reducing sodium dodecyl sulfate–polyacrylamide gel electrophoresis (SDS‐PAGE); other protein separation methods such native, nonreducing, and 2D methods may be substituted. Proteins are loaded onto polyvinylidene difluoride (PVDF) membranes via wet electrophoretic transfer, and cellular components are then quantified via antibody recognition of epitopes and subsequent coupling to a luminescent readout. This protocol contains instructions for quantification of total protein and phosphoprotein content with near‐infrared imaging of fluorophore‐conjugated secondary antibodies. Near‐infrared imaging (LI‐COR Odyssey or equivalent) has a broad dynamic range amenable to densitometry quantification in LI‐COR Image Studio Lite or other software package (e.g., NIH ImageJ; see Internet Resources). The Alternate Protocol describes visualization of blots by chemiluminescence imaging.

We describe a method for stimulating adherent BMDMs with depleted zymosan, a β‐glucan preparation that binds the hemi‐ITAM‐containing receptor Dectin‐1 (Underhill, 2003). This representative cell‐activating stimulus can be coupled with pharmacological, transcriptional, or genetic disruption of tyrosine kinase function to test the contribution of these kinases to cell signaling. Alternative receptor ligation or inhibition of analog‐sensitive Csk (Csk^AS^) by the small molecule 3‐IB‐PP1 can be used as an alternative to Dectin‐1 clustering. In the latter approach, 3‐IB‐PP1 inhibits a sensitized form of Csk, the tyrosine kinase that negatively regulates the Src family tyrosine kinases (SFKs). When Csk^AS^ is inhibited, SFKs become activated and initiate signaling through many pathways (see Background Information; Brian et al., 2019; Freedman et al., 2015; Schoenborn, Tan, Zhang, Shokat, & Weiss, 2011; Tan et al., 2014). Dectin‐1 ligation is a useful positive control for myeloid cell activation via tyrosine kinase–dependent signaling (Freedman et al., 2015; Goodridge et al., 2011), but the choice of controls for a given experiment should reflect the cell and pathway of interest. Where appropriate, we include adaptations applicable to lymphocytes and other cells in suspension.

### Materials

Cells of interest (e.g., BMDMs)
Cell culture medium (e.g., Dulbecco's modified Eagle medium [DMEM‐10]; see recipe)
Phosphate‐buffered saline (PBS), without calcium or magnesium (e.g., Corning, MT21031CV)
Cell dissociation buffer (e.g., Gibco, 13151014)
Polarization agent
Depleted zymosan (e.g., Sigma, Z4250; for preparation see Underhill, 2003)
Kinase inhibitor (e.g., PP2; Thermo, PHZ1223)
3‐IB‐PP1 (e.g., Millipore, 529598)
SDS sample buffer (see recipe)
1 M dithiothreitol (DTT; e.g., Fisher Scientific, BP172‐5)
1× running buffer (see recipe)
1× transfer buffer (see recipe)
Tris‐acetate protein gel (e.g., Fisher Scientific, EA03585BOX)
Molecular weight marker (e.g., Bio‐Rad, 161‐0394)
Methanol (e.g., Honeywell, AH230‐4)
Total protein stain (e.g., LI‐COR, 926‐11021)
Total protein wash (see recipe)
Total protein removal solution (see recipe)
1× tris‐buffered saline (TBS; see recipe)
Blocking buffer (see recipe or purchase from commercial source; e.g., LI‐COR, 927‐50003)
Primary antibody
Primary diluent (see recipe)
1× TBS containing Tween‐20 (TBST; see recipe)
Secondary antibody appropriate for primary antibody (e.g., LI‐COR)
Secondary diluent (see recipe)

Cell culture incubator
150‐mm^2^ non‐tissue culture‐treated plate
Midspeed centrifuge (e.g., Sorvall Legend XTR)
Hemocytometer
6‐well non‐tissue culture‐treated plate (e.g., Corning, 351146)
Cell scraper (e.g., Corning, 353085)
1.5 ml snap‐lock microcentrifuge tubes (e.g., Eppendorf, 022363611)
Sonicator (e.g., Diagenode Bioruptor Pico or other small‐capacity bath or probe sonicator)
Thermomixer (e.g., Eppendorf, 2231000033) or heat block
Refrigerated microcentrifuge (e.g., Eppendorf, 5415R)
Electrophoresis and wet transfer running unit (e.g., Invitrogen, EI0002)
Power source (e.g., Invitrogen, PS0300)
Sponges (e.g., Invitrogen, EI9052)
Immobilon‐FL PVDF membrane (e.g, Millipore, IPFL00010)
Filter paper (e.g., GE Healthcare, 30306132)
Nontranslucent incubation box (e.g., LI‐COR, 929‐96310)
Orbital rocker
Near‐infrared imaging system (e.g., LI‐COR Odyssey)
Computer running LI‐COR Image Study Lite or similar software and spreadsheet analysis software (e.g., Microsoft Excel)

### Stimulation and cell lysis

1 Grow BMDMs according to published protocols (Freedman et al., 2015; Zhu, Brdicka, Katsumoto, Lin, & Weiss, 2008). Seed BMDMs on 150‐mm^2^ plates.
  The type of plate or flask and the preparation procedure depends on the model system.
2 On day 6 or 7, detach adherent BMDMs: Aspirate medium and wash once with PBS. Dispense 8 ml cell dissociation buffer. Return cells to incubator for ≤15 min, tapping to see if cells separate from the plastic. Remove cells by repeatedly pipetting cell dissociation buffer over the plate surface and rinsing once with fresh PBS. Centrifuge cells 5 min at 400 × *g*, 4°C. Resuspend cells in DMEM‐10 and count.
3 Transfer cells to 6‐well plate (10^6^ cells per 2 ml DMEM‐10 with or without polarization agents).
  Each well within a single plate can contain a separate genotype, polarization condition, or treatment condition within a single time point.
  The number of cells required for analysis will depend on the quality of the primary antibody and the expression level and modification stoichiometry of the target protein. If more lysate is needed, multiple identical wells can be prepared, or a larger plate can be used. Duplicate wells are preferable if a centrifugation step is part of subsequent cell treatment (e.g., depleted zymosan).
4 Rest BMDMs overnight at 37°C in a 10% CO_2_ incubator.
  Adherent cells such as BMDMs should be seeded onto plates and rested overnight prior to stimulation. Cells cultured in suspension (e.g., Jurkat T cells or mast cells) can be stimulated in sterile 1.5‐ml polypropylene tubes and require less resting—on the order of minutes (T cells) to hours (mast cells), optimized to minimize basal signaling.
5 Prewarm a midspeed centrifuge to 37°C by spinning at 6000 × *g*.
  Warming time will vary depending on the centrifuge.
6 Prepare 0.5 ml depleted zymosan in DMEM‐10 with or without kinase inhibitor (e.g., 20 µM PP2, a SFK inhibitor) for each stimulation and time point.
  Owing to poor aqueous solubility, most inhibitors will need to be diluted from a concentrated stock solution in dimethyl sulfoxide or other solvent. Appropriate controls, including vehicle‐only treatments, are therefore important. For some inhibitors, efficacy may be increased if cells are pretreated prior to stimulation; this must be optimized for the particular cell or condition. Most kinase inhibitors, including PP2, inhibit multiple kinases (see Background Information). For each inhibitor it is important to be familiar with these potential side effects and to use a minimum effective dose to avoid inhibiting secondary pathways (Knight & Shokat, 2005).
7 Gently remove 1.5 ml DMEM‐10 supernatant from each well, and return plates to incubators for at least 10 min to re‐equilibrate the temperature.
8 Gently apply 0.5 ml sonicated and washed depleted zymosan or 3‐IB‐PP1 with or without kinase inhibitor (or alternative stimulation/perturbation). Quickly but gently place plates in the prewarmed centrifuge, and pulse spin 30 s at 5000 × *g* to synchronize deposition of depleted zymosan particles onto cells.
  For short incubation times plates may remain in the stationary, warmed centrifuge. For longer time points plates should be gently returned to the incubator to ensure temperature stability.
9 Stop signaling at the desired time point by placing the plate on ice. Quickly aspirate supernatant.
  Time points will likely need to be optimized. Many receptors will induce phosphorylation cascades within a few seconds and peak by 5 to 30 min. To analyze longer‐term changes in signaling or transcription or feedback‐induced changes in cell activation, time scales on the order of hours or days may be appropriate. To determine the immediate roles of a particular tyrosine kinase, it is best to start with a shorter time window to evaluate direct or immediate downstream effects.
10 Lyse cells by adding 200 to 400 µl SDS sample buffer and DTT to 50 mM. Scrape cells off plate, and incubate at 37°C for 5 min. Pipette cell lysates into labeled 1.5‐ml snap‐lock tubes.
  The snap‐lock feature prevents tubes from popping open during later boiling steps.
11 Lyse cells and shear DNA by sonication with chilling (e.g., three times for 1 min at 50% duty cycle with a chilled Diagenode Bioruptor Pico).
12 Incubate samples 15 min at ≥99°C. Microcentrifuge samples 30 s at 10,000 × *g*, room temperature.
  Samples can be refrigerated or frozen until further analysis: 4°C for short‐term, −20°C for medium‐term, or −80°C for long‐term storage.
  If samples are refrigerated or frozen before blotting, warm them to ≥37°C, pulse centrifuge, and remix before running SDS‐PAGE. If sample quality deteriorates, fresh DTT may be added and samples reboiled. Samples can usually undergo freeze‐thaw cycles several times before their quality deteriorates.

### Gel electrophoresis and wet transfer

**Figure 1:**
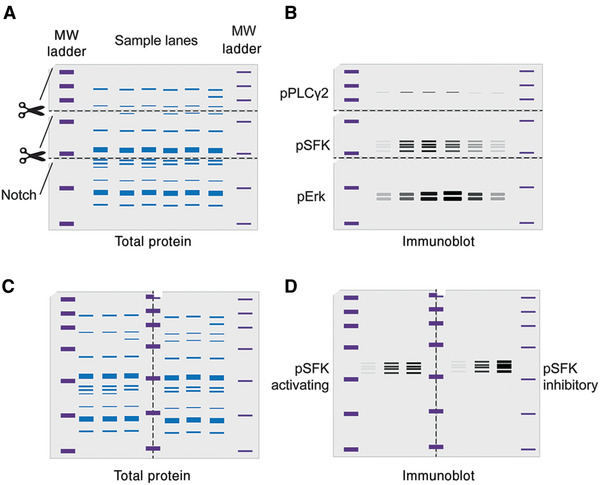
Suggested cutting/notching technique for blots. (**A**) Using molecular weight (MW) ladders as a guide, membranes can be cut to increase antibody multiplexing. Notches are shown to ensure correct membrane orientation. (**B**) Theoretical staining of a membrane using different antibodies on different membrane sections. (**C**) Using MW markers as a guide, membranes can be cut vertically to increase sample processing. Notches are shown to ensure correct membrane orientation.

13 Prepare running buffer and transfer buffer.
  This protocol has been optimized for Tris‐acetate gels. See manufacturer instructions for running conditions for other types of gels.
14 Prepare gels by removing the comb from the gel and rinsing each lane with running buffer to remove gel fragments.
15 Load ∼2.5 × 10^4^ cell equivalents into each lane, taking care not to puncture the gel. For best results load the same volume in each well. Load the left‐most lane with molecular weight marker. Include positive and negative controls on each gel to facilitate quantification across blots. Load unused wells with SDS sample buffer.
  The loading strategy is appropriate for immunoblots from whole‐cell lysates for moderately expressed proteins using near‐infrared imaging. Optimization may be needed if probing extremely abundant or rare proteins, events, or immunoprecipitates.
  If blots will later be cut horizontally, straight cutting may be facilitated by loading two‐ to three‐times diluted (to indicate left to right directionality) molecular weight marker in the right‐most lane. If blots will be cut vertically, it is advisable to use marker lanes between segments (Fig. 1).

16 Fill electrophoresis module with running buffer, and apply constant voltage (150 V) until the dye front has migrated out of the gel or the desired separation has occurred (∼80 min).
  The time and voltage will depend on the size of the target protein(s) and the gel and buffer system being used.
17 While the gel is running, prepare transfer apparatus. Cut Immobilon‐FL PVDF membrane to size, and rinse membrane in methanol to activate. Rinse three times with distilled water. Place membrane in transfer buffer.
  Many types of PVDF and nitrocellulose membranes are available commercially. Ensure that the membrane is suitable for use with fluorescent secondary antibodies (e.g., “FL” designation) to minimize background. If chemiluminescence detection is more accessible, refer to the Alternate Protocol for the appropriate membrane.
  This protocol uses wet transfer methodology, which generally produces the highest‐quality results across a wide range of molecular weights. See manufacturer's instructions for semi‐dry transfer buffers and setup.
18 Assemble transfer apparatus according to the manufacturer's instructions. Place one corner of the membrane on top of the gel. Slowly place the opposite corner of the membrane onto gel, and lower the rest of the membrane onto the gel, taking care to avoid trapping bubbles. Orient transfer with the membrane on the positive (anode) side and gel on the negative (cathode) side.
19 Fill inner and outer chambers of the apparatus with cold transfer buffer. Place on ice or in a cold room.
  Chilling is critical for minimizing heat deformation of the gel during transfer.
20 Transfer 1.75 hr at low voltage (30 V) on ice.
  The timing and voltage may need to be optimized.
21 Remove membrane from the apparatus, and dry in between two sheets of clean filter paper to fix proteins onto the membrane.
  Handle the membrane minimally, wearing clean gloves and using clean tweezers to touch only the edges. We recommend notching the blot in the upper corner with a clean razor blade to delineate front versus back and left versus right directionality.

### Total protein staining

22 Place membrane in an incubation box, and activate by soaking 1 min in methanol. Discard methanol and rinse three times for 30 s each with water.
23 Add 5 ml total protein stain. Rock 5 min in the dark at room temperature.
  Total protein stain is a more robust method for quantification than a single protein reference such as GAPDH, β‐actin, or Erk1/2, although these typical loading controls are still useful in figures as visual aids. For quantification of phosphoproteins, it may be more appropriate to use an antibody specific to the protein of interest (modified vs unmodified) rather than a pan‐total protein stain.
24 Discard total protein stain, and wash two times for 30 s each with total protein wash.
25 Rinse membrane three times with water, and image gel with a near‐infrared imaging system.
26 Rinse membrane briefly in water. Replace solution with total protein removal solution. Rock 5 min in the dark at room temperature.

### Immunoblotting

27 Discard solution and place membrane in methanol.
28 If cutting membrane into segments of different molecular weights, place membrane on clean filter paper, and cut with clean scissors or razor blade. Return to methanol.
29 Discard methanol and rinse three times for 30 s each with water.
30 Discard water. Rock 2 min in 5 to 10 ml TBS at room temperature.
31 Discard TBS and add 5 to 10 ml blocking buffer. Rock 1 hr at room temperature in the dark.
  Blocking buffer may be purchased from commercial vendors (e.g., LI‐COR) or made in‐house with bovine serum albumin (BSA) or powdered milk. In our experience buffer purchased from LI‐COR works very well.
  Powdered milk is generally a more effective blocking agent than BSA but may affect detection of phosphoproteins due to high background from the phosphoprotein casein.
  A blot may be left at 4°C in blocking solution overnight or even over a few days. For consistent results, however, blocking time should be kept relatively consistent.
32 Dilute primary antibody in 4 to 6 ml (depending on the size of the container) of 1:1 blocking buffer:primary diluent.
  Optimal dilution will vary by antibody. Antibodies of different species can be combined in the same solution if multiple emission wavelength channels are available (e.g., rabbit‐derived anti‐phospho‐Erk1/2 combined with mouse‐derived anti‐Erk1/2 imaged in separate channels).
  If an antibody on an uncut gel is sufficiently specific as to produce a single band in a particular experimental condition, multiple antibodies of the same color and/or species may be pooled when detecting proteins separated by molecular weight. If there is any doubt about specificity, cut blots instead of combining antibodies.
  An optimal dilution of primary antibody should be evaluated by titration for each cell type and stimulation condition, but a good starting range is 1:1000 to 1:5000. With near‐infrared imaging, primary antibodies can typically be diluted 2 to 20 times more than recommended by the manufacturer.
  Most primary antibody solutions can be used multiple times if stored at 4°C in sodium azide (NaN_3_).
33 Discard blocking buffer, and add diluted primary antibody. Mix overnight at 4°C.
  In some cases primary antibodies specific for abundant proteins or moieties can be applied for 1 hr at room temperature. For best reproducibility, keep a consistent incubation time.
34 Remove and store diluted primary antibody. Wash membrane three times for 5 min each with TBST.
35 Dilute secondary antibody in 1:1 blocking buffer:secondary diluent.
  Dilution of the secondary antibody should be optimized for each condition. For near‐infrared detection, secondary antibodies are typically used at 1:10,000 to 1:20,000. When combining secondary antibodies to image two proteins on a near‐infrared imager, use the brighter, 800‐nm channel for lower‐abundance or phosphorylated proteins and the dimmer, 700‐nm channel for higher‐abundance proteins.
36 Incubate 1 hr at room temperature.
37 Wash three times for 5 min each with TBST.
38 Wash 2 min with TBS to remove residual Tween‐20.
39 Dry membrane between two sheets of clean filter paper.
40 Image membrane protein‐side down using a near‐infrared imager.
  In rare cases total protein stain can decrease the signal in subsequent blots. If troubleshooting is necessary, consider skipping steps 22 to 26.

### Quantification of total protein by densitometry

41 Select appropriate fluorescence channel in the right‐hand Display tab of Image Studio Lite.
42 In the Analysis tab, select Draw Rectangle. Draw a rectangle around the entire lane of interest (test darkest lane first). Rotate or resize box using the graph in the right‐hand Profile tab. Move box to the left‐most lane, duplicate, and drag boxes to the other lanes. Adjust each box, if necessary, using the Profile tab.
  The software will take box size into account, but it is best to keep the boxes uniform. Some lysates may have a very dark cluster of bands overlaid on a lighter lane background. It may be useful to exclude these major bands from the quantification to avoid signal skewing by a few undefined proteins. Boxes in this case can be drawn from the top of the lane and end before these bands. If the blot has imperfections, boxes may be redrawn to exclude them.
  If bands for total protein or specific stains are round or bleed between lanes, less lysate should be loaded into future blots, if possible.
  Boxes may be rotated to account for lane slanting, but the gel image itself should not be rotated prior to densitometry analysis.
43 In the Background pane, select User Defined for background quantification.
44 Draw a small box in between two lanes with representative background fluorescence. In the Background tab, select Assign Shape to apply this box for background subtraction.
45 Export data from the Shapes tab into Microsoft Excel or other spreadsheet manager. Use the background‐corrected “Signal” column for data normalization and graphing.

### Quantification of immunoblots (repeat for each protein of interest)

46 Select appropriate fluorescence channel in the right‐hand Display tab of Image Studio Lite.
47 In the Analysis tab, select Add Rectangle. Place a box on the image by clicking near the darkest band of interest. Rotate or resize the box using the graph in the right‐hand Profile tab. Move box to the left‐most lane, duplicate, and drag boxes to the other lanes. Adjust each box, if necessary, using the Profile tab.
  Boxes may be rotated to account for lane slanting, but the gel image itself should not be rotated prior to densitometry analysis.
48 In the Background pane, select Median for background quantification. Adjust borders to top/bottom or right/left, and choose the background box size.
  The directionality and size of background boxes will depend on the shape of the band being quantified, how well separated the lanes are, whether there are nonspecific or unidentified bands above or below the band of interest, and whether the lanes are generally higher in the background than the space in between lanes.
49 Export data from the Shapes tab into Microsoft Excel or other spreadsheet manager. Use the background‐corrected “Signal” column for data normalization and graphing.
  Report the abundance of a protein or modification relative to the total protein stain within the same lane. It may be appropriate to report post‐translational modifications relative to a total protein immunoblot for the protein of interest. These two analytical approaches will reflect an overall dose in the cell population versus a more stoichiometry‐like assessment of the degree of modification within the existing protein.
  It may also be useful to perform a second normalization step relative to a reference (e.g., time = 0, wild‐type, or unpolarized) value. This will obscure basal differences between treatment groups but clarify differences in kinetic response to the cell treatment or perturbation.

## VISUALIZING IMMUNOBLOTS USING CHEMILUMINESCENCE

Horseradish peroxidase (HRP)‐conjugated antibodies in conjunction with chemiluminescence imaging is another common approach to visualizing immunoblots. In contrast to direct dye conjugation in near‐infrared imaging, HRP‐adsorbed blots are developed by addition of an HRP substrate (a luminol/enhancer mixture) that generates a chemiluminescent signal from HRP‐conjugated secondary antibodies. Although this method can in some cases be quantitative, the dynamic range is typically narrower than in near‐infrared imaging, and it is easy to over‐ or under‐produce signal in this indirect method. To achieve the best signal, gel loading, antibody dose, and substrate choice should be optimized. An advantage of this approach is that the HRP enzyme can be efficiently inactivated and the blot reprobed with a different set of antibodies.

### Additional materials (also see Basic Protocol 1)

HRP‐conjugated secondary antibody (e.g., Southern Biotech)
SuperSignal West Femto Maximum Sensitivity Substrate (e.g., Thermo Scientific, 34096)

Plastic wrap
Luminescence imager

1 Complete steps 1 to 22 and steps 28 to 38 of Basic Protocol 1 using HRP‐conjugated secondary antibody for step 35.
  As in Basic Protocol 1, the dilution of each antibody must be optimized. For chemiluminescence imaging, the primary antibody (Basic Protocol 1 step 32) will typically be diluted according to manufacturer's instructions. HRP‐conjugated secondary antibodies are typically diluted 1:100,000.
  The HRP enzyme is sensitive to azide (N_3_), so it is important to wash blots thoroughly after incubation with primary antibody.
  As described in Basic Protocol 1, with proper controls, antibodies can be combined if their output is highly specific and/or clearly identifiable by molecular weight.
  Unlike near‐infrared fluorescence imaging, HRP antibody–bound membranes can be treated with NaN_3_ and frozen to inactivate the HRP enzyme. Blots can then be reprobed with different antibodies (Freedman et al., 2015). This is especially useful if the second set of antibodies is derived from a different species to prevent fresh HRP secondary antibody binding to the previously adsorbed primary antibody.
  Buffers are marketed for stripping blotting antibodies from membranes. In our experience this can work but often not uniformly across the membrane surface, which can limit the fidelity of quantification. We recommend performing multiple blots rather than stripping if HRP activation is not sufficient to resolve different sets of proteins.
2 Prepare substrate working solution by combining equal amounts of peroxide and enhancer solutions (from SuperSignal kit). Place membrane on a piece of plastic wrap, and pipet a minimum volume of substrate working solution onto the surface of the blot. Tilt membrane to thoroughly coat, and watch for bands to develop, following manufacturer's instructions.
  HRP working solution is stable for ∼8 hr and can be reused.
3 Cover membrane in clear plastic, and smooth to remove bubbles. Image using a luminescence imaging system.
  The linear range for chemiluminescence detection is much narrower than that of near‐infrared imaging, so quantification should be approached with more caution. Check linearity using serial dilution of lysates or samples. If bands appear black on the edges and white in the middle (or the reverse, depending on the image display mode) or football‐ rather than bar‐shaped, the image is likely overexposed. A range of substrate solutions with differential sensitivity (Femto, Pico, Atto) is available, so the substrate can be optimized for the abundance of the blotting target. The incubation time and lane loading can also be optimized.

## Basic Protocol 2 ENRICHING TARGET PROTEINS AND ISOLATION OF PROTEIN COMPLEXES BY IMMUNOPRECIPITATION

**Figure 2:**
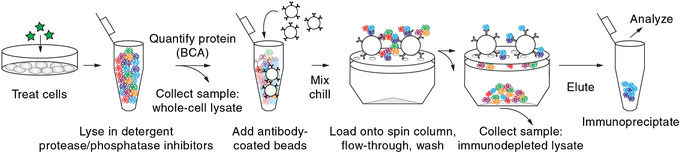
Key steps in co‐immunoprecipitation. Cells are lysed with a nondenaturing detergent in the presence of protease and phosphatase inhibitors. Protein quantification ensures comparability across different samples. Antibodies and beads are added to the lysate to enrich specific proteins or protein complexes. Noninteracting or weakly interacting proteins are removed by washing. The enriched proteins and complexes are then eluted for further analysis.

First described in the 1970s (Kessler, 1975), immunoprecipitation is a common method for separating proteins from cell lysates in denaturing or nondenaturing conditions. It has been further refined for protein purification, enrichment of low‐abundance species, and identification of protein complexes (co‐immunoprecipitation). As a tool for studying cell signaling, immunoprecipitation typically starts with preparation of an antibody–bead complex (noncovalent in this protocol, covalent in the Support Protocol). These antibody‐coated beads are then mixed with cell lysates and gently tumbled under conditions that maximize protein capture but minimize protein degradation and further post‐translational modification. The protein–antibody–bead complex is then collected, and the (co‐)immunoprecipitated proteins are eluted for analysis (Fig. 2).

This protocol describes immunoprecipitation of a target protein from cell lysate. In the absence of the usual loading controls available in whole‐cell lysate (described in Basic Protocol 1), a protein content normalization step is essential for quantitative analysis. The immunoprecipitation time, detergent, and salt content will determine the extent of interacting protein co‐immunoprecipitation. The composition of the immunoprecipitate can then be probed by blotting for pan‐phosphotyrosine, specific phosphorylation sites, total protein, or other epitopes to reveal protein–protein interactions and post‐translational modifications that follow cell perturbation (as in Basic Protocol 1). The immunoprecipitates can also be subjected to phosphoproteomics to identify unknown proteins or targeted mass spectrometry to quantify specific peptides and post‐translational modifications. A targeted approach is described in Basic Protocol 3.

### Materials

Protein G (or A or other functionalized) Sepharose beads (e.g., GE Healthcare, 17061801)
PBS (e.g., Corning, MT21031CV)
Immunoprecipitation antibody
Protease and phosphatase inhibitor (e.g., Sigma‐Aldrich, MSSAFE‐5VL)
n‐Dodecyl β‐d‐maltoside (lauryl maltoside) lysis buffer (see recipe)
Cells of interest (see Basic Protocol 1)
Normal serum (e.g., Jackson ImmunoResearch)
Immunoprecipitation elution buffer (see recipe)
Bicinchoninic acid (BCA) assay kit (e.g., Thermo Scientific, 23225)
NP‐40 alternative wash buffer (see recipe)

Refrigerated microcentrifuge (e.g., Eppendorf, 5415R)
Vortex
Tube rotator
Cell scraper (e.g., Corning, 353085)
1.5‐ml LoBind (low‐adsorption) microcentrifuge tubes (e.g., Eppendorf, 022431081)
Sonicator (e.g., Diagenode Bioruptor Pico or other small‐capacity bath or probe sonicator)
Micro‐Bio spin chromatography column (e.g., Bio‐Rad, 732‐6204)

### Cell stimulation and lysis

1 Collect 4 to 5 µl protein G Sepharose beads per 10^6^ cells by microcentrifuging 2 min at 500 × *g*, room temperature. Carefully remove supernatant, and replace with PBS. Pulse vortex, collect beads, and repeat wash two times.
  The choice of immunoprecipitation antibody (subclass, host species, and prior functionalization) and any functionalization of the cells or lysates will determine the optimal bead adsorption modality (protein G, protein A, streptavidin, other). As an example, see Table 1 for specificities of proteins G and A.

**Table 1:** Antibody Binding Specificities of Proteins G and A

Species	Antibody subclass	Protein G binding	Protein A binding
Guinea Pig	IgG_1_	Medium binding	Strong binding
Hamster		Medium binding	Low binding
Human	IgG_1_	Strong binding	Strong binding
	IgG_2_	Strong binding	Strong binding
	IgG_3_	Strong binding	Weak or low binding
	IgG	Strong binding	Strong binding
	IgM	Weak or low binding	Variable
Monkey		Strong binding	Strong binding
Mouse	IgG_1_	Strong binding	Low binding
	IgG_2a_	Strong binding	Strong binding
	IgG_2_	Moderate binding	Moderate binding
	IgG_3_	Moderate binding	Medium binding
	IgM	Weak or low binding	Variable
Rabbit		Moderate binding	Strong binding
Rat	IgG_1_	Low binding	Weak or low binding
	IgG_2a_	Strong binding	Weak or low binding
	IgG_2b_	Medium binding	Weak or low binding
	IgG_3_	Medium binding	Weak or low binding

Adapted from *Affinity Chromatography. Vol. 1: Antibodies* (see Internet Resources).

2 Prebind immunoprecipitation antibody to beads by incubating 1 to 2 µg antibody per 40 × 10^6^ cells with beads. Rotate beads at least 2 hr at room temperature prior to use.
  In this protocol the immunoprecipitation antibody will co‐elute with the target protein, possibly yielding dark bands caused by the antibody heavy chain at ∼50 to 70 kDa and the light chain at 25 kDa (Harlow & Lane, 1988). Secondary antibodies may even react across species owing to the sheer abundance of the antibody bands. For optimal visualization and quantification of proteins close to either molecular weight, it is advisable to use covalent conjugation to prevent antibody elution from the beads. One such method is described in the Support Protocol.
3 Add protease and phosphatase inhibitors to an aliquot of chilled lauryl maltoside lysis buffer. Protect from light and keep on ice until use.
  We have had the most success using lauryl maltoside detergent for cell lysis and immunoprecipitation. Other detergents, such as NP‐40 alternative, can decrease the number of loosely interacting proteins that co‐immunoprecipitate with the target.
  Detergent choice should be tailored to experimental needs (Firestein, Gabriel, McInnes, & O'Dell, 2017; Johnson, 2013).
  NP‐40 alternative is less expensive than lauryl maltoside and can be substituted in wash steps.
4 Prepare and treat cells as described in steps 1 to 9 of Basic Protocol 1.
  If stimulating a large number of cells, they can be rested on larger plates (e.g., 150 mm^2^).
5 Quench signaling by washing cells two times with ice‐cold PBS and placing plates on ice.
6 Lyse cells by adding 50 µl lauryl maltoside lysis buffer per 10^6^ cells.
7 Scrape plates to lift cells, and collect in sterile 1.5‐ml LoBind tubes.
8 Sonicate cells (5 min total, 50% duty cycle in a Diagenode Bioruptor Pico) to disrupt membranes, break up protein aggregates, and shear DNA.
9 Centrifuge lysate 15 min at 15,000 × *g* in a chilled microcentrifuge to remove insoluble material.
  Micro‐ultracentrifugation at 90,000 × g for 15 min may be used in place of sonication and benchtop microcentrifugation.

### Immunoprecipitation

10 Preclear samples by adding 50 µl protein G Sepharose beads and 10 µl normal serum per 1 ml lysate. Rotate 30 min at 4°C.
  The normal serum should be species matched to the immunoprecipitation antibody. Also, tailor the preclearance mode to the bead/functionalization.
11 Collect beads by centrifuging 2 min at 500 × *g* in a chilled microcentrifuge, and transfer precleared supernatant to new tube. Keep all tubes on ice.
12 Mix 50 µl whole‐cell lysate with 50 µl immunoprecipitation elution buffer for later immunoblot.
13 Remove 50 µl lysate for BCA assay (see manufacturer's instructions). Calculate protein concentration in each sample, and aliquot corrected volumes of lysate into LoBind tubes.
  This BCA step is important for quantitative analysis because immunoprecipitates lack the usual loading controls, namely housekeeping proteins and/or a true total protein stain. Users can expect to recover 50 µg protein per 10^6^ cells. However, this will vary based on cell type and lysis.
14 Wash antibody–beads two times with PBS, discarding supernatant. Resuspend antibody–beads in lauryl maltoside lysis buffer, and portion evenly among lysate samples.
15 Tumble lysates and beads 2 hr at 4°C to immunoprecipitate protein of interest.
  Immunoprecipitation time can be optimized. Longer incubation times (e.g., overnight) may allow protein complexes to dissociate or increase the efficiency of pulldown, depending on the sample. Longer incubation times can also lead to protease‐mediated sample degradation.
16 Apply samples to spin columns. Centrifuge 2 min at 450 × *g*, 4°C. Mix 50 µl flow‐through with 50 µl immunoprecipitation elution buffer for immunodepleted blotting samples.
  If spin columns are unavailable, collect beads by centrifugation, and remove supernatant via pipetting. With this approach, bead loss and less‐efficient washing may decrease the reproducibility of later quantification steps, but these issues can be minimized with conservative removal of supernatant and increased number or volume of wash steps.
  For enzyme assays or other applications, do not apply samples to spin columns or elute with denaturing (SDS‐containing) immunoprecipitation elution buffer.
17 Wash beads and column five times with 1 ml NP‐40 alternative wash buffer, centrifuging 2 min at 450 × *g*, 4°C, and discarding flow‐through between washes.
  To avoid disrupting protein complexes, it may be desirable to continue using lauryl maltoside or other detergent instead of NP‐40 alternative.
18 Elute immunoprecipitated protein by applying enough immunoprecipitation elution buffer to cover the beads in the spin column. Incubate 15 min at room temperature. Elute 5 min at 10,000 × *g*, 4°C, in a microcentrifuge.
19 Incubate lysate and immunoprecipitate samples 5 min at ≥99°C. Centrifuge samples 1 min at 10,000 × *g*, 4°C. Handle and store gel samples as described in step 12 of Basic Protocol 1.
20 To assess the efficiency of immunoprecipitation and the general stoichiometry of co‐immunoprecipitated protein binding, run immunoblots with whole‐cell and immunodepleted lysates, as described in steps 13 to 46 of Basic Protocol 1. Assess immunoprecipitates by immunoblot, skipping the total protein stain in steps 22 to 26 of Basic Protocol 1.
  Immunoprecipitates can be further probed by immunoblotting as described in Basic Protocol 1 or by targeted mass spectrometry as described in Basic Protocol 3. These samples can also be analyzed using unbiased mass spectrometry.

## COVALENT CONJUGATION OF ANTIBODIES TO FUNCTIONALIZED BEADS

In some cases, it is best to conjugate immunoprecipitation antibodies covalently to immunoprecipitation beads rather than co‐eluting antibodies with the immunoprecipitate samples. For immunoblotting analysis (Basic Protocol 1), covalently conjugated antibody–beads complexes produce cleaner images, facilitating visualization and quantification of proteins comigrating with the heavy and light chains. Covalent conjugation is also ideal for subsequent mass spectrometry analysis (Basic Protocol 3) in that resulting immunoprecipitates can be desalted and run directly without further purification of proteins or detergents that would otherwise harm the mass spectrometer. In spite of these advantages, covalent conjugation tends to be used selectively because of the increased investment of time and reagents.

### Materials

Protein G (or A or other functionalized) Sepharose beads (e.g., GE Healthcare, 17061801)
PBS (e.g., Corning, MT21031CV)
Immunoprecipitation antibody
Dimethyl pimelimidate (DMP; e.g., Thermo Scientific, 21666)
0.15 M sodium borate, pH 9.0 (e.g., Millipore, SX03551)
0.2 M ethanolamine, pH 8.0 (e.g., Fisher Scientific, M251‐1)
1 M glycine, pH 3.0 (e.g., Fisher Scientific, BP381‐1)
NaN_3_

15‐ml conical tubes (e.g., Falcon, 14‐959‐70C)
Centrifuge
Tube rotator

*NOTE*: If this protocol will be followed by mass spectrometry analysis, wear a face mask and gloves for all steps to minimize keratin contamination.

1 Collect enough protein G Sepharose (or alternative) beads for each conjugation.
  Each vial of DMP yields 10 ml crosslinking solution, enough for one conjugation reaction with 1 ml beads. The antibody and beads should be titrated for the specific immunoprecipitation. Start with 2 µl antibody and 50 µl beads per 8 × 10^6^ macrophages.
  As with immunoblotting, start by doubling the number of lymphocytes or other cells in suspension to ensure adequate material for immunoprecipitation.
2 Wash beads twice with PBS by centrifuging 30 s at 1000 × *g*, room temperature.
3 Resuspend in PBS.
4 Add immunoprecipitation antibody, and tumble 1 hr at room temperature.
5 Prepare 10 ml of 20 mM DMP in 0.15 M sodium borate, pH 9.0, per 1 ml beads.
  CAUTION: DMP is highly light sensitive. Keep covered. Check pH after DMP addition.
6 Wash beads twice with 10 ml of 0.15 M sodium borate, pH 9.0.
7 Resuspend beads in 20 mM DMP in 0.15 M sodium borate, pH 9.0.
8 Mix beads and DMP 30 min at room temperature on a rotator.
9 Collect beads and remove DMP solution. Quench by adding 10 ml of 0.2 M ethanolamine, pH 8.0. Incubate 2 hr at room temperature with gentle mixing.
10 Spin beads down by briefly centrifuging and remove ethanolamine. Elute unbound antibody by incubating two times for 10 min each with 1 M glycine, pH 3.0, at room temperature.
11 Wash beads with PBS.
12 Resuspend beads in PBS with 0.02% (w/v) NaN_3_.
  Test antibody conjugation prior to large‐scale immunoprecipitation experiments.

## Basic Protocol 3 QUANTIFYING PROTEINS AND POST‐TRANSLATIONAL MODIFICATIONS BY TARGETED MASS SPECTROMETRY

Commercial antibodies are not available for every protein epitope and post‐translational modification. Antibodies may be raised against custom sequences, but this process is costly and at times problematic. Mass spectrometry is a valuable tool for detecting changes in protein homeostasis and identifying novel sites of modification prior to investing in antibody generation. We present a protocol for quantifying phosphorylation on an immunoprecipitated protein via targeted liquid chromatography–tandem mass spectrometry (LC‐MS/MS), using parallel reaction monitoring (PRM) on a high‐resolution mass spectrometer. Traditional, data‐dependent acquisition triggers fragmentation of the top N ions in a MS1 scan (the first component of MS/MS), effectively surveying the most abundant populations of ions. PRM instead uses precursor ion selection to trigger fragmentation of modified peptides of interest and creating full‐scan MS2 spectra, improving selectivity, sensitivity, and signal‐to‐noise ratios (Rauniyar, 2015). This protocol may be adapted for other epitopes or modifications and for kinase (or other) activity assays for probing modification of a target site in cells, lysates, or recombinant proteins.

**Figure 3:**
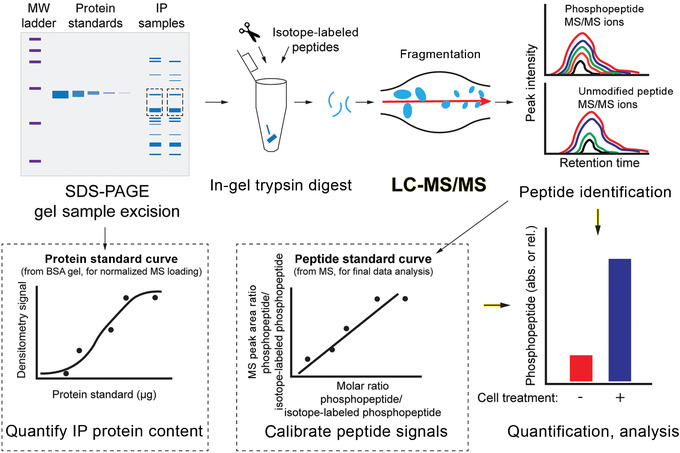
Key steps for in‐gel digestion and targeted LC‐MS/MS. Immunoprecipitated samples are resolved via gel electrophoresis and excised based on molecular weight. Protein standards (e.g., bovine serum albumin [BSA]) of known concentration are used to quantify gel loading to standardize loading of immunoprecipitate samples onto the LC‐MS/MS and normalizing later peptide or phosphopeptide analysis. A reference quantity of isotope‐labeled reference peptide, to be used as an internal standard, is added to the gel fragments. The gel sample/peptide mixture is subjected to protease digest and targeted LC‐MS/MS analysis. By comparing the endogenous and isotope‐labeled phosphopeptide peaks and peptide standard curves, molar and relative quantities of phosphorylated and unphosphorylated peptides can be calculated.

We describe steps to ensure accurate peptide identification and quantification using a heavy isotope–labeled internal standard. Prior to beginning proteolytic in‐gel digestion, a BCA assay is used to quantify the total protein concentration in cell lysates, a critical step for normalizing phosphopeptide levels across samples. Subsequently, protein concentrations are determined using a standard curve of titrated BSA, quantified by densitometry after SDS‐PAGE. This ensures that phosphopeptide quantification can be expressed as a concentration ratio relative to the amount of protein subjected to tryptic digest. Finally, a stable isotope–labeled reference peptide is spiked into the immunoprecipitate prior to proteolytic digestion. Peptide concentrations can then be definitively identified and placed on an absolute scale via a peptide standard curve (Fig. 3).

### Materials

Immunoprecipitated samples (see Basic Protocol 2)
SDS sample buffer (see recipe)
BCA protein assay kit (e.g., Thermo Scientific, 23225)
Tris‐acetate protein gel (e.g., Fisher Scientific, EA03585BOX)
SimplyBlue SafeStain (e.g., Invitrogen, LC6065)
100 mM ammonium bicarbonate (e.g., JT Baker, 300301)
Acetonitrile, HPLC grade (e.g., Fisher Scientific, A955‐4)
DTT (e.g., Fisher Scientific, BP172)
Iodoacetamide (e.g., Sigma‐Aldrich, I1149)
Trypsin digest solution (see recipe)
Reference peptides (e.g., Sigma‐Aldrich)
Custom‐synthesized ^13^C,^15^N‐heavy isotope amino acid–labeled reference peptide corresponding to digested phosphopeptide of interest
Custom‐synthesized unlabeled phosphorylated peptide standard corresponding to digested phosphopeptide of interest
CaCl_2_ (e.g., Honeywell‐Fluka, C1016100G)
Formic acid (e.g., Fisher Scientific, A117‐50)
Water, HPLC grade (e.g., Fisher Scientific, W6‐4)
Desalting wash solvent (see recipe)
Trifluoroacetic acid
Desalting wetting solvent (see recipe)
Desalting elution solvent (see recipe)
Calibration curve buffer (see recipe)
HPLC buffer A (see recipe)
HPLC buffer B (see recipe)

Centrifugal filter column (e.g., Millipore, UFC500324)
Electrophoresis system (e.g., Invitrogen, EI0002)
Near‐infrared imaging system (e.g., LI‐COR Odyssey)
Razor blade
1.5‐ml LoBind microcentrifuge tubes (e.g., Eppendorf, 022431081)
Vortex
Microcentrifuge
Variable temperature incubator
Vacuum concentrator (e.g., SpeedVac; Thermo Scientific, SPD140DDA)
C18 reverse‐phase extraction disk (e.g., 3M, 2240/2340)
18‐G needle
HPLC system (e.g., Thermo Easy‐nLC 1000)
Silica PicoTip Emitter Column, 100‐µm ID, 75‐cm final length (e.g., New Objective)
ReproSil‐Pur C18 AQ LC column (packed in‐house)
Orbitrap Fusion Tribrid Mass Spectrometer (e.g., Thermo Fisher)
Computer running Skyline Targeted Mass Spec software (see Internet Resources)

### Quantification of protein content

1 Perform an immunoblot (Basic Protocol 1) with immunoprecipitated samples (Basic Protocol 2). Serially dilute samples in SDS sample buffer (e.g., 1:1, 1:2, 1:10).
2 Quantify amount of immunoprecipitated protein for each sample via densitometry (Basic Protocol 1). Create a standard curve using signals from the serially diluted lanes to calculate the relative amount of precipitated protein in each sample.
3 Concentrate equal amounts of immunoprecipitated protein sample with as much lysate as possible to ensure detection of potentially rare peptides via LC‐MS/MS and using centrifugal filter columns according to manufacturer's instructions. Store eluent at −80°C indefinitely.

### Gel electrophoresis and quantification

For the following steps, wear a face mask and gloves to minimize keratin contamination during gel loading, excision, reduction, and alkylation.

**Figure 4:**
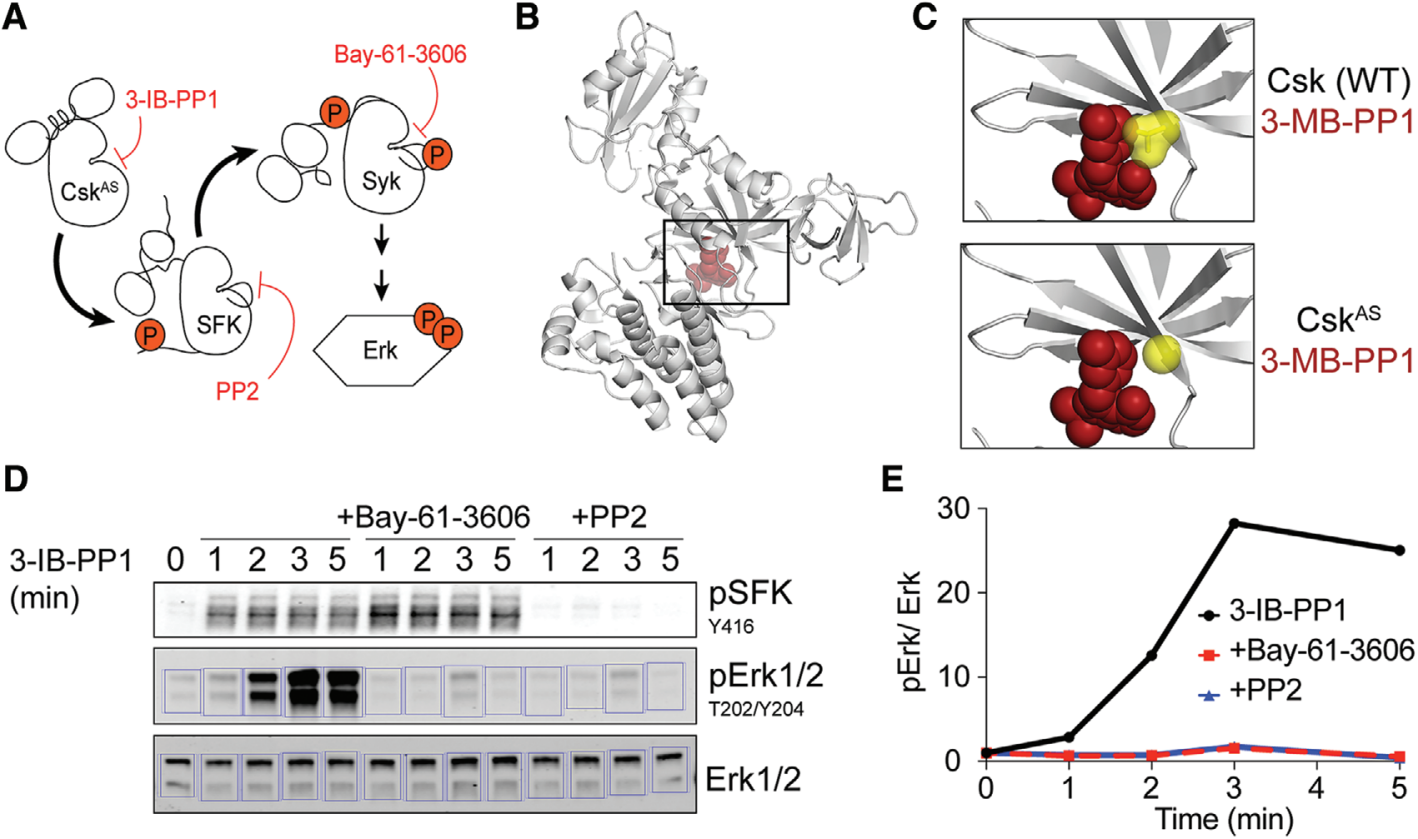
Analog‐sensitive and other pharmacological methods for inhibiting kinases. (**A**) The analog‐sensitive kinase Csk^AS^ is inhibited by the bulky PP1 analog 3‐IB‐PP1, leading to Src family kinase (SFK) activation and subsequent activation of Syk tyrosine kinase. These activating effects can be blocked with small‐molecule inhibitors of downstream kinases, such as PP2 (Src family) and BAY‐61‐3606 (Syk). (**B**) Crystal structure of Csk (gray; PDB ID: 1K9A; Ogawa et al., 2002) modeled with the ATP binding site occupied by a bulky analog of the kinase inhibitor PP1 (3‐MB‐PP1; red). The box outlines the zoomed images in the following panel. Image rendering and modeling was performed in Pymol. (**C**) Zoomed views of wild‐type (WT) and (modeled) Csk^AS^ with the gatekeeper (residue 266) highlighted in yellow. Threonine‐to‐glycine substitution of the gatekeeper residue (T266G) enlarges the ATP binding pocket, accommodating the bulky inhibitor. Wild‐type Csk and endogenous kinases are not as sensitive to bulky inhibitor analogs (Freedman et al., 2015; Tan et al., 2014). (**D**) Immunoblot illustrating SFK and Erk1/2 phosphorylation following treatment of Csk^AS^ macrophages with 3‐IB‐PP1 in the presence or absence of Syk and SFK inhibitors. Background is calculated from boxes to the left and right of the lane. Imaging was performed with a LI‐COR Odyssey, and densitometry was performed in Image Studio Lite. (**E**) Quantification of pErk normalized to Erk levels for the immunoblot shown in D.

4 Prepare BSA protein quantification standards at 0.05 to 20 µg total per lane.
  The concentrations of the BSA standards will depend on the protein concentration of the concentrated immunoprecipitated samples and may need to be optimized.
5 Load gel with immunoprecipitated samples and BSA standards. Resolve samples by SDS‐PAGE (see Basic Protocol 1).
6 Stain gel with SimplyBlue SafeStain in a clean container according to the manufacturer's instructions.
  An alternative stain may be used if it is compatible with mass spectrometry.
7 Remove stain by washing two times for 1 hr each with distilled water.
8 Image gel on an appropriate imaging system.
  Use a compatible imager for the appropriate total protein staining solution.
9 Generate a standard curve via densitometry analysis of the BSA bands, as described in Basic Protocol 1 and shown in Figure 4. Use this curve to quantify the amount of experimental sample in each lane.
  We typically quantify the total protein in each lane across all molecular weights. It may be more appropriate with recombinant proteins to quantify only the band of interest in each immunoprecipitate.

10 Using a fresh, clean razor blade for each sample, excise a sample of gel corresponding to the desired protein (molecular weight range), and place into 1.5‐ml LoBind tubes.

### Reduction and alkylation

11 Cut gel samples into small (∼2 mm) pieces. Wash gel fragments three times for 15 min each by submerging in ∼100 µl (depending on gel fragment size) of a 1:1 mixture of 100 mM aqueous ammonium bicarbonate:acetonitrile. Mix by vortexing prior to each incubation.
12 Remove final wash, and incubate 1 min in 100% acetonitrile, until gel pieces turn opaque. Collect fragments by briefly centrifuging in a microcentrifuge and discard acetonitrile.
13 Submerge gel fragments in an aqueous solution of 10 mM DTT/50 mM ammonium bicarbonate. Incubate 1 hr at 56°C. Pulse spin in a microcentrifuge and remove supernatant.
  Make DTT solution fresh by dissolving DTT into 50 mM ammonium bicarbonate.
14 Submerge fragments in an aqueous solution of 55 mM iodoacetamide/50 mM ammonium bicarbonate. Incubate 30 min at room temperature in the dark. Pulse spin in a microcentrifuge and remove supernatant.
  Make iodoacetamide solution fresh by dissolving iodoacetamide in 50 mM ammonium bicarbonate.
15 Wash gel fragments twice with a 1:1 mixture of 100 mM ammonium bicarbonate:acetonitrile.
16 Remove solution and dry fragments by incubating 1 min in 100% acetonitrile.

### In‐gel protease digest

17 Remove acetonitrile and cover with trypsin digest solution (see Shevchenko, Wilm, Vorm, & Mann, 1996) spiked with ^13^C,^15^N‐heavy isotope amino acid–labeled reference peptides. Incubate 15 min on ice.
  The precise concentration of heavy‐labeled reference peptide spiked in during in‐gel digest will depend on the final yield of protein extracted from the gel. This can be estimated by densitometry from the BSA curve generated after SDS‐PAGE. However, we suggest doing a trial run to ensure the spiked‐in reference peptide is not orders of magnitude higher or lower in concentration than the peptides of interest. Trypsin, which cuts at lysine and arginine residues (Ma, Tang, & Lai, 2005), is often the protease of choice. If the distribution of lysine and arginine around the sequence of interest is suboptimal for LC‐MS/MS detection, another protease such as chymotrypsin, LysC, or LysN (Giansanti, Tsiatsiani, Low, & Heck, 2016) may be used to optimize digested peptide m/z and facilitate detection.
18 Remove excess trypsin digest solution, and cover gel fragments with an aqueous solution of 50 mM ammonium bicarbonate/5 mM CaCl_2_. Digest samples 16 hr at 37°C.

### Peptide extraction

19 Collect gel fragments in a microcentrifuge by pulse spinning. Remove supernatant and place in a new LoBind tube.
20 Extract peptides from gel fragments in a minimum volume of 50% (v/v) acetonitrile/0.3% (v/v) formic acid in HPLC‐grade water. Pulse vortex and incubate 15 min at room temperature.
21 Transfer peptide‐containing supernatant to the LoBind tube from step 19.
22 Submerge fragments in 80% (v/v) acetonitrile/0.3% (v/v) formic acid in HPLC‐grade water. Pulse vortex and incubate 15 min at room temperature.
23 Pool second extraction in the LoBind tube with the previous supernatant. Store at −80°C indefinitely.
24 Remove solvent by vacuum concentration (e.g., SpeedVac), and store at −80°C indefinitely.

### Peptide desalting

25 Assemble desalting tips by punching two holes from C18 reverse‐phase extraction material with an 18‐G needle and expelling into a 200‐μl pipet tip using a clean capillary tube (Rappsilber, Ishihama, & Mann, 2003; Rappsilber, Mann, & Ishihama, 2007).
26 Add 60 µl desalting wash solvent to vacuum‐dried peptide samples. Vortex 45 s and centrifuge 1 min at 3000 × *g*, room temperature. Add more trifluoroacetic acid if necessary to adjust pH ≤3.
27 Wet stage tips with 60 µl desalting wetting solvent. Centrifuge 2 min at 450 × *g*, room temperature.
28 Discard solvent and apply acidified samples to stage tip. Centrifuge 2 min at 450 × *g*, room temperature.
29 Wash stage tip two times with 60 µl desalting wash solvent. Centrifuge 2 min at 450 × *g*, room temperature.
30 Place stage tip into new LoBind tube, and elute peptides with 60 µl desalting elution solvent. Centrifuge 2 min at 450 × *g*, room temperature.
31 Remove solvent by vacuum concentration (e.g., SpeedVac), and store at −80°C indefinitely.

### Preparation of calibration curve samples

32 Dilute 1000 fmol heavy isotope–labeled phosphorylated peptide standard in calibration curve buffer into several LoBind tubes.
33 To create a calibration curve to quantify the amount phosphorylated peptide, add increasing concentrations of unlabeled phosphorylated peptide standard so that the molar ratio of unlabeled phosphorylated peptide standard:heavy‐labeled phosphorylated peptide standard spans 0.1 to 1.5.
  The precise molar ratios will depend on the assay and the amount of phosphorylated peptide in each sample.
34 Dilute 1000 fmol phosphorylated peptide standard in calibration curve buffer into several LoBind tubes.
35 To create a calibration curve to quantify the ratio of phosphorylated and unphosphorylated peptide in each sample, add increasing amounts of unphosphorylated peptide standard such that the molar ratio ranges from 0.05 to 1.5.
  The peptide ratios used in this calibration curve will depend on the stoichiometry of tyrosine phosphorylation and may need to be adjusted depending on the rarity of phosphorylation for a given phosphorylation site.
36 Submit calibration curve samples and in‐gel digested samples for LC‐MS/MS (steps 37 to 42).

### LC‐MS/MS

37 Load a 75‐cm × 100‐µm silica PicoTip Emitter column for nanospray with ReproSil‐Pur 1.9‐mm C18 AQ.
38 Mount loaded PicoTip Emitter column in a nanospray source in line with an Orbitrap Fusion with 2.1 kV spray voltage in the positive mode and heated capillary maintained at 275°C.
39 Set up a tripartite peptide elution program decreasing the fraction of HPLC buffer A and increasing the fraction of HPLC buffer B with a 300 nl/min flow rate:
  5% to 10% HPLC buffer B over 5 min
  10% to 16% HPLC buffer B over 40 min
  16% to 26% HPLC buffer B over 5 min.
  The elution program should be optimized depending on the m/z and hydrophobicity of the target peptide and desired resolution. This step presents a general starting point in three gradient stages.
40 Define an acquisition method comprising a full scan and PRM to detect singly, doubly, and triply charged precursor ions without scheduling. Set the full scan event to employ a 380 to 1500 *m/z* selection, an Orbitrap resolution of 60,000 (at *m/z* 200), a target automatic gain control (AGC) value of 4 × 10^5^, and maximum ion injection time of 50 ms. Set the PRM scan to employ an Orbitrap resolution of 30,000 (at *m/z* 200) and a target AGC value of 5 × 10^4^ and/or maximum ion injection time of 54 ms to ensure that enough fragment ions are captured for MS/MS detection.
  The acquisition method and scan events will vary depending on the chemical composition of the targets and the number of peptides analyzed in a given experiment. When there are few peptides, method development can be simplified by monitoring selected precursor ions for the duration of the chromatography gradient. For quantitative experiments, a selected peptide must be surveyed and an MS2 acquired at least 10 times across the extracted ion chromatogram (EIC). Scheduling may be used when the number of possible peptide precursors is >20 in order to capture 10 MS2 scans across a peptide chromatogram.
  Quantification can be performed using MS1 or MS2 (the two components of MS/MS) EICs in the Skyline software package (see Internet Resources). A full spectrum scan facilitates assessment of dynamic range issues, co‐eluting peptides, and complexity of the sample and acts as an additional validation of accurate mass for the peptide of interest. The m/z range defined above surveys all ions with a charge >1. At the above resolution EICs can be used to uniquely identify co‐eluting peptides with small m/z differences so they can be fragmented individually for identification by MS2. Fill time is simply the time we allow the ions to fill the chamber.
41 Set the MS2 quadrupole isolation window to 1.6 *m/z*. Perform fragmentation with a higher‐energy collision‐induced dissociation (HCD) of 30%, and collect an MS2 scan from 100 to 1000 *m/z*.
  HCD will depend on peptide chemistry and phosphorylation site sequence context, so it will have to be optimized (Diedrich, Pinto, & Yates, 2013).
42 Collect PRM data in centroid mode, and export for quantification.
  Centroid data acquisition decreases file size.

### Data analysis using Skyline

#### Configuration

43 Analyze data in the Skyline Targeted Mass Spec program (see Internet Resources; MacLean et al., 2010; Pino et al., 2020). Open the Skyline Start Page, and select Blank Document and Save As.
44 Select the Settings tab, and locate Peptide Settings. Input parameters to reflect the experimental settings.
  Peptide Settings parameters can vary depending on a variety of factors, including digestion enzyme, peptide length, peptide modifications, cleavage sites, and type of internal standard.
45 In the Settings tab locate Transition Settings.
  Transition Settings parameters vary according to a variety of factors—y and b ion series, precursor ions, charge states, mass accuracy, and method (targeted or untargeted) of LC‐MS/MS acquisition.
46 Navigate to the Edit tab, then Insert and Peptides. Enter the phosphorylated peptide sequences and select Insert.
  The Targets list on the left Skyline panel will be populated.

#### Importing and inspecting standard raw data

47 Import raw mass spectrometer files into Skyline by navigating to File, Import, and Results. Choose Add single injection replicates in files and select OK, which will prompt the Import Results Files to show raw standard curve data files. Upload the selected files by choosing Open, followed by Do Not Remove when the option to remove the naming prefix appears. Confirm and close the window by selecting OK.
48 Using raw files generated from standards (e.g., heavy isotope and light isotope phosphopeptides), inspect the chromatographic traces for quality control.
  If chromatographic peaks have a non‐Gaussian peak shape, the samples and standards should be rerun for quality assurance. Inconsistent LC retention times could reflect inadequate chromatographic resolution. Phosphopeptide transition ions should be chosen based on relative signal intensity of their EIC and selected for ions that are representative of larger y or b ions in the peptide fragmentation series. For example, a 10‐mer peptide may fragment to yield a y^9^ ion, but the y^8^ ion may exhibit an EIC that is higher intensity and should thus be selected for quantification. Peptide sequence and length also affect selection of transitions for peptide validation and quantification.
49 Manually inspect each peptide‐extracted product ion chromatogram.
  Isotope labeling should not affect the retention time of otherwise identical peptides. Selected transitions should exhibit proportional distributions.

#### Analyzing PRM data from samples

50 Import raw sample files into Skyline as described in step 47.
51 Inspect chromatographic traces, retention times, and fragmentation patterns of heavy and light phosphorylated peptides in each sample.

#### Generating a calibration curve

52 Define concentrations of external standards. To do so, go to the View tab, and select Document Grid.
53 Go to the top‐left Reports dropdown menu, and select Replicates from the Reports list.
54 Define the standard raw files as Sample Type Standard, and specify their Analyte Concentrations. Select Unknown for sample raw files.
55 To view the calibration curve, go to the View menu, and select Calibration Curve.
56 Access Reports from the Document Grid, and select Peptide Quantification. Prepare a report in the Export tab, enter the file name, and click OK.
57 Normalize raw quantifications for each sample using the total protein amount used for in‐gel digestion.

## REAGENTS AND SOLUTIONS

### Blocking buffer

3% (w/v) BSA
25 mM Tris base
125 mM NaCl
0.02% (w/v) NaN_3_
Adjust pH to 8.0 with NaOH
Store at 4°C for up to 6 months

### Calibration curve buffer

5% (v/v) acetonitrile
0.1% (v/v) trifluoroacetic acid
Water, HPLC grade
Store at 4°C for up to 1 year

### Desalting elution solvent

40% (v/v) acetonitrile
0.1% (v/v) trifluoroacetic acid
Water, HPLC grade
Store at 4°C for up to 1 year

### Desalting wash solvent

2% (v/v) acetonitrile
0.1% (v/v) trifluoroacetic acid
Water, HPLC grade
Store at 4°C for up to 1 year

### Desalting wetting solvent

20% (v/v) acetonitrile
0.1% (v/v) trifluoroacetic acid
Water, HPLC grade
Store at 4°C for up to 1 year

### DMEM‐10

DMEM containing 4.5 g/L glucose and glutamine (e.g., Corning, 10‐017‐CM)
10% (v/v) fetal bovine serum
0.11 mg/ml sodium pyruvate
2 mM penicillin/streptomycin
2 mM l‐glutamine
Store at 4°C for up to 1 year

### HPLC buffer A

0.1% (v/v) formic acid
Water, HPLC grade
Store at 4°C for up to 1 year

### HPLC buffer B

0.1% (v/v) formic acid
99.9% (v/v) acetonitrile, HPLC grade
Store at 4°C for up to 1 year

### Immunoprecipitation elution buffer

125 mM Tris base
10% (v/v) glycerol
5% (v/v) 2‐mercaptoethanol
25% (w/v) SDS
0.1% (w/v) bromophenol blue
Store at 4°C for up to 6 months

### Lauryl maltoside lysis buffer

1% (w/v) lauryl maltoside
150 mM NaCl
0.01% (w/v) NaN_3_
Store at 4°C for up to 1 year
  Immediately before use add protease and phosphatase inhibitors.

### NP‐40 alternative wash buffer

1% (v/v) NP‐40 alternative (e.g., Thermo Fisher Scientific, 49‐201‐850ML)
150 mM NaCl
10 mM Tris·HCl, pH 7.6
0.01% (w/v) NaN_3_
Store at 4°C for up to 1 year
  Immediately before use add protease and phosphatase inhibitors.

### Primary diluent

1× TBS (see recipe)
0.2% (v/v) Tween‐20
0.02% (w/v) NaN_3_
Store at 4°C for up to 6 months

### Running buffer, 20×

1 M tricine
1 M Tris base
2% (w/v) SDS
Store at 4°C for several months
  Dilute to 1× working solution before use.

### SDS sample buffer

128 mM Tris base
10% (v/v) glycerol
4% (w/v) SDS
0.1% (w/v) bromophenol blue
Adjust pH to 6.8 with 1 M HCl
Store at 4°C for up to 1 year
  Immediately before use add DTT to 50 mM.

### Secondary diluent

1× TBS (see recipe)
0.2% (v/v) Tween‐20
0.04% (w/v) SDS
0.02% (v/v) NaN_3_
Store at 4°C for up to 6 months

### TBS, 20×

2.5 M NaCl
0.5 M Tris base
Filter sterilize
Store at room temperature for up to 6 months
  Dilute to 1× working solution before use.

### TBST, 20×

4 M NaCl
0.5 M Tris base
1% (v/v) Tween‐20
Filter sterilize
Store at room temperature for up to 6 months
  Dilute to 1× working solution before use.

### Total protein removal solution

0.1 M NaOH
30% (v/v) methanol
Store at 4°C for up to 6 months

### Total protein wash

6.7% (v/v) acetic acid
30% (v/v) methanol
Store at 4°C for up to 6 months

### Transfer buffer, 20×

500 mM bicine
500 mM Bis Tris
20 mM EDTA
Apply gentle heat to dissolve
Store at 4°C for up to several months
  On the day of transfer, dilute to 1× working solution in chilled water, supplement with 10% (v/v) methanol, and keep cold.

### Trypsin digest solution

50 mM NH_4_HCO_3_
5 mM CaCl_2_
5 ng/µl trypsin, sequencing grade (e.g., Promega, V5111)
Store at −80°C indefinitely
  Add heavy isotope–labeled reference peptide as necessary for experiment.

## COMMENTARY

### Background Information

Tyrosine kinases are important regulators of immune cell activation, proliferation, and survival (Bryan & Rajapaksa, 2018). Transfer of the terminal phosphate of ATP to a tyrosine residue on a protein substrate results in changes in conformation and protein–protein interaction that act as signals to direct cellular function (Lemmon & Schlessinger, 2010). The growth, survival, and proliferation functions of tyrosine kinases are important in all cells. Immune cells employ peculiar binding motifs, alternative expression of kinase family members, and additional receptor families for additional functionalities such as phagocytosis, antigen‐specific signaling, and polarization. In lymphocytes, hematopoietic SFKs initiate signaling downstream of T and B cell receptors by phosphorylating immunoreceptor tyrosine‐based activation motifs (ITAMs), which leads to activation of the tandem SH2–containing tyrosine kinases Syk and Zap‐70. Together, these tyrosine kinases activate FAK family tyrosine kinases (FAK, Pyk2) and Tec family tyrosine kinases (Btk, Itk, Tec; Hwang et al., 2020). Parallel pathways are activated upon Fc receptor engagement in myeloid and NK cells (Bradshaw, 2010; Cox & Greenberg, 2001; Freedman et al., 2015; Futosi & Mocsai, 2016; Lowell, 2011).

Janus kinase (JAK) activation downstream of receptor tyrosine kinases is critical for activation of signal transducer and activator of transcription (STAT) proteins that mediate growth, differentiation, and polarization (Villarino, Kanno, & O'Shea, 2017). Other receptor tyrosine kinases such as Flt3, c‐Kit, and Tyro/Axl/Mer control cell survival, differentiation, and many other essential functions of immune cells (Masson & Ronnstrand, 2009; Rothlin, Carrera‐Silva, Bosurgi, & Ghosh, 2015). Despite the many inputs that engage tyrosine kinases and an intense research focus on the tyrosine kinases involved in immune activation, we are still discovering elements of the interactions and dynamics of tyrosine kinases with profound effects on immune regulation (Brian et al., 2019; Courtney et al., 2017; Freedman et al., 2015; Hwang et al., 2020; Salter et al., 2018). Understanding the dynamics, kinetics, substrates, and scaffolding interactions of tyrosine kinases is critical to developing therapeutics that modulate immune function (Roschewski et al., 2020; Salter et al., 2018; Solouki, August, & Huang, 2019).

Numerous tools exist for studying the actions of tyrosine kinases in immune cells, including genetic methods such as siRNA knockdown, CRISPR/Cas9‐based gene editing, small‐molecule inhibitors, and chemical–genetic designer kinase–inhibitor pairs. Each approach has advantages and disadvantages with regard to specificity, temporal control, and likelihood of triggering compensatory mechanisms (Table 2).

**Table 2:** Tools for Studying Tyrosine Kinase and Other Immune Cell Signaling

Method	Advantages	Disadvantages
Small‐molecule inhibitors	Inexpensive, rapid inhibition, no genetic compensation	Poor selectivity, low solubility of inhibitors
Knockout models	Specificity, no barrier to studies in vivo	Transcriptional feedback leading to altered signaling, time/labor intensive, expensive to maintain
Analog‐sensitive kinases	Rapid kinase inhibition, no transcriptional feedback, easily portable and robust	High degree of investment for design and screening
Immunoblotting	Detection of low‐abundance proteins, wide compatibility	Low throughput, depends on availability of validated, site‐specific antibodies
Nontargeted proteomics	Broad in scope, unbiased by model, reveals novel sites from heterogeneous samples	Limited quantification, limited sensitivity for low‐abundance proteins and rare events
Targeted proteomics	Precise quantification, even of low‐abundance proteins or phosphorylation events, does not rely on availability of antibodies	Significant assay development, limited scope

Genetic methods are attractive options for studying kinase function because of their inherent specificity and stability. While knockout gene editing strategies are valuable because they offer complete disruption of kinase signaling, siRNAs offer inducible control over kinase signaling disruption and are especially useful when knocking out a given kinase is lethal or maturation‐impairing to a cell type or animal. siRNAs and genetic knockouts are routinely used to investigate the roles of kinases in immune cells. For instance, mice in which the SFK Lyn has been knocked out have become important models of autoimmune disease after studies revealed the importance of Lyn as a negative regulator of B cell and dendritic cell activation (Brodie, Infantino, Low, & Tarlinton, 2018; Scapini, Pereira, Zhang, & Lowell, 2009). Tyrosine kinase knockouts can also be coupled to *Cre*‐*lox* and FLP‐FRT systems for cell‐specific knockout (Lamagna, Hu, DeFranco, & Lowell, 2014; Lamagna, Scapini, van Ziffle, DeFranco, & Lowell, 2013). The advent of CRISPR‐Cas9 gene editing has facilitated the substitution of specific amino acid residues in knockin models, allowing researchers to dissect novel elements of tyrosine kinase function (Harder et al., 2001). The major drawback of knockout and knockdown models for studying kinase signaling is that cells often develop compensatory mechanisms for coping with loss of the given kinase. These feedback (or, in cell lines, evolutionary) effects may mask the normal signaling contributions and scaffolding interactions of the kinase of interest (El‐Brolosy & Stainier, 2017; Peng, 2019).

Small‐molecule inhibitors have facilitated the study of kinases in many aspects of immune activation. Kinase inhibitors generally function by competing with ATP for access to the active site, preventing substrate phosphorylation (Davies, Reddy, Caivano, & Cohen, 2000). Although a large number of compounds are marketed for inhibition of specific kinases, caution should be used when choosing an inhibitor and interpreting its effects on signaling. ATP binding sites are highly conserved across the kinome (Manning, Whyte, Martinez, Hunter, & Sudarsanam, 2002), and most inhibitors target multiple kinases, either within a family or in different branches of the kinome (Fabian et al., 2005). Researchers should familiarize themselves with these off‐target effects and use the lowest effective concentration of inhibitor to disfavor weaker binding interactions. Furthermore, many kinase inhibitors are poorly soluble in aqueous buffers, necessitating formulation for experiments in vivo or pretreatment for experiments in vitro (Eckstein et al., 2014; Herbrink, Schellens, Beijnen, & Nuijen, 2016). A final consideration when working with ATP‐mimetic inhibitors is that these inhibitors typically bind and may even induce the active conformation of the kinase. This can lead to a paradoxical increase in typical readouts of kinase activation (e.g., phosphorylation of the activation loop tyrosine) and may even ultimately promote signaling due to release of autoinhibition. Careful controls (e.g., phosphorylation of inhibitory/activating sites on the kinase and direct substrates) should be probed along with downstream readouts of cell activation. Ultimately, however, small‐molecule inhibitors for many kinases are well characterized and commercially available and require little up‐front investment of time or resources. Moreover, a pharmacological approach can uniquely enable the study of transient effects with high kinetic fidelity and minimal regulatory compensation. Inhibitors are thus powerful tools for dissecting kinase contribution to immune activation.

Chemical–genetic methods for studying kinase signaling in immune cells combine the specificity of gene editing with the temporal control of small‐molecule inhibitors. In one approach kinases are sensitized to a bulky analog of an ATP competitive kinase inhibitor by substituting a smaller amino acid side chain for the usual aliphatic, polar, or bulky gatekeeper residue (Lopez, Kliegman, & Shokat, 2014). Since the gatekeeper is not directly involved in ATP binding, the “analog‐sensitive” kinase retains kinase activity until the designer inhibitor is added (Bishop et al., 2000). This chemical–genetic approach can be used in transfected cells or incorporated into the genome of a model animal as a transgene or knockin. Since endogenous kinases have more occlusive gatekeeper residues, the engineered kinase–inhibitor pair is much more specific than traditional kinase inhibition (Fig. 4). Importantly, analog‐sensitive kinase inhibition has the additional advantage over genetic or siRNA knockout approaches in that normal kinase function in the absence of inhibitor will allow direct comparison of cells pre‐ and post‐treatment. This real‐time component also minimizes the likelihood of compensatory transcriptional changes and other adaptations in primary cells or animals and selective pressure and evolution in cell lines. This approach has been used to identify the specific roles for Zap‐70 in T cell activation and Csk in T cell and macrophage activation, but the approach can be applied to other kinases as well (Freedman et al., 2015; Levin, Zhang, Kadlecek, Shokat, & Weiss, 2008; Tan et al., 2014). Furthermore, although many kinase inhibitors blunt signaling, some kinases such as Csk have paradoxical negative regulatory functions. Inhibition of Csk^AS^ with 3‐IB‐PP1 leads to robust SFK activation (Freedman et al., 2015; Tan et al., 2014). Inhibition of these negative regulatory kinases can be used as potent stimuli of cellular signaling and can be combined with other kinase inhibitors to tease apart kinase contribution to cellular activation and protein dynamics (Brian et al., 2019).

Although some information can be gleaned from unbiased total protein and pan‐phosphotyrosine detection methods, immunoblotting is typically most effective when applied as a targeted, relatively low‐throughput process, requiring antibodies raised against unique peptide sequences or sites of post‐translational modification. The best antibodies have minimal cross‐reactivity with other molecules in the cell. Small‐volume, higher‐throughput apparatuses are available, but these systems are less amenable to combining antibodies and resolving multiple species in a single blot. Despite these caveats, immunoblotting remains a robust, sensitive, and adaptable technique (Kurien & Scofield, 2015). If epitope‐specific antibodies are unavailable, immunoblotting can be combined with immunoprecipitation. For example, total protein immunoprecipitation can be followed with a pan‐phosphotyrosine blot, and molecular weight can be used to infer the identity of phosphorylated protein (Freedman et al., 2015). Alternatively, cyanogen bromide fragmentation (Thofte et al., 2018) can resolve phosphorylation of individual sites on multiply phosphorylated proteins.

Freed of the requirement for specific antibodies, LC‐MS/MS is an excellent exploratory technique for quantifying poorly studied proteins and sites of post‐translational modification. This method is especially useful for multiply modified proteins that cannot easily be probed by blotting. Advances in LC‐MS/MS have allowed researchers to quantify tyrosine phosphorylation via targeted and unbiased approaches (Dekker et al., 2018; Hu, Noble, & Wolf‐Yadlin, 2016; Liu & Chance, 2014). Proteomics approaches use databases to identify enzyme‐digested peptides following resolution by LC‐MS/MS. Unbiased LC‐MS/MS can identify novel sites of phosphorylation in a cell lysate but may miss low‐abundance peptides. In contrast, targeted approaches using isotope‐labeled reference peptides are highly sensitive and can be used to quantify abundance or novel sites of post‐translational modification in cell lysates and in vitro kinase assays using recombinant or purified proteins.

Together, these protocols describe powerful tools for investigating tyrosine kinase and other cell modulatory signaling. The methods range from general and flexible when reagents are available (immunoblotting) to more focused (co‐immunoprecipitation and LC‐MS/MS). Together, they constitute a process for dissecting the kinetics and dynamics of signaling pathway activation, protein–protein interaction, and novel tyrosine phosphorylation that are essential for understanding how the many inputs that engage tyrosine kinases are involved in immune activation, allowing researchers to develop tools that modulate immune function by directing kinase signaling.

### Critical Parameters

*Basic Protocol* *1*: A million cells lysed in 400 µl lysis buffer should yield enough protein for analysis with near‐infrared‐conjugated secondary antibodies and an appropriate imager (e.g., LI‐COR Odyssey). We have found that this ratio works well for macrophages, but the ratio may need to be adjusted (∼doubled) for smaller cells, such as primary T and B cells, Jurkat cells, and mast cells, depending on the protein being analyzed. It is possible to use fewer cells, but the lysis buffer volume should be scaled to maintain comparable protein concentrations. Before beginning, primary antibodies should be validated to ensure specificity to the desired protein being probed.

*Basic Protocol* *2*: For each condition a large number of cells (8–40 × 10^6^) is required to ensure immunoprecipitation of sufficient protein for subsequent analysis. Stringency of the buffer, incubation, and wash conditions should be optimized so that only specific, biologically relevant protein–protein interactions are preserved. Stringency of co‐immunoprecipitation can be assessed by blotting for nonassociated and loosely associated proteins in immunoprecipitates. To increase the stringency of immunoprecipitation, researchers can screen different lysis detergents and increase the salt concentration (over the typical 150 mM) in the wash buffer. It is also critical to keep lysis buffers, wash buffers, and beads cold to prevent phosphatase and protease activity.

*Basic Protocol* *3*: When working with gels prior to protease digest, it is critical to avoid keratin contamination. Be sure to wear gloves, a face mask, and a laboratory coat, working to limit breathing or leaning over the gel as much as possible. Surfaces, tools, and instruments should be thoroughly cleaned with tissue wipes and 70% ethanol prior to use. Iodoacetamide and DTT should be portioned and dissolved in appropriate buffers immediately before use to prevent degradation from light. Buffer conditions, acquisition method, and scan events will vary depending on the chemical properties of the target peptides and samples and will require extensive method development using synthetic peptide standards (isotope‐labeled and unlabeled). Data analysis will also change based on the peptides being studied.

### Troubleshooting

*Basic Protocol* *1*: See Internet Resources (e.g., Good Westerns gone bad, LI‐COR) for information on common issues and troubleshooting techniques for immunoblotting.

*Basic Protocol* *2*: Whereas primary antibodies for immunoblotting most likely recognize denatured proteins, antibodies for immunoprecipitation must recognize proteins in their native conformation. Optimization with different antibody clones may be needed. Buffers and incubation periods should be optimized to ensure immunoprecipitation stringency is desired.

*Basic Protocol* *3*: If no peptides are detected following LC‐MS/MS, researchers should ensure the immunoprecipitation process is optimized to enrich the desired protein. If no phosphorylated peptides are detected, ensure the LC‐MS/MS method is first optimized to detect both phosphorylated and unphosphorylated reference peptides. We use trypsin as a protease for in‐gel digestion in this protocol; however, trypsin digestion may not yield peptides suitable for MS/MS detection, necessitating the use of other enzymes with different amino acid preferences for peptide digestion.

### Understanding Results

*Basic Protocol* *1*: Immunoblotting should reveal distinct bands for each probed protein at the correct molecular weight. Immunoblotting with optimal primary and secondary antibody dilutions with near‐infrared secondary antibodies should prevent signal saturation.

*Basic Protocol* *2*: Immunoprecipitation should enrich protein complexes of interacting proteins. The robustness of immunoprecipitation can be assessed by immunoblotting for proteins that are not anticipated to interact with the immunoprecipitated protein.

*Basic Protocol* *3*: Targeted LC‐MS/MS for protein phosphorylation should identify both phosphorylated and nonphosphorylated peptides that can be quantified and normalized to gel loading based on detection of a protein (BSA) standard curve (shown in Fig. 4) and with calibration curve samples based on the ratio of heavy isotope.

### Time Considerations

*Basic Protocol* *1*: Cell stimulations will vary by the signaling pathway and cell type of interest and can range from seconds to days. The time required for SDS‐PAGE and immunoblotting will depend on the particular system being used: roughly 1 to 2 hr for SDS‐PAGE and 2 hr for wet transfer systems described here. However, dry transfer systems can take substantially less time. Primary antibody incubation will depend on the particular antibody being used and usually requires 1 hr at room temperature or overnight at 4°C. Secondary antibody incubation with near‐infrared antibodies lasts 1 hr plus an additional 30 min for total washing.

*Basic Protocol* *2*: If antibody–bead conjugation is being used (Support Protocol), the process should be completed at least the day before beginning immunoprecipitation. The success of antibody–bead conjugation should be verified by a separate experiment. Antibody–bead conjugation takes roughly 4 to 5 hr. Cell stimulation and lysis prior to immunoprecipitation will vary by the signaling pathway and cell type of interest and can range from seconds to days. The BCA assay to measure protein content after lysis takes ∼1 hr. Immunoprecipitation incubations can vary by primary antibody and lysis buffers and typically range from 1 hr to overnight.

*Basic Protocol* *3*: The entire process for Basic Protocol 3 requires several days, although there are several places in which samples can be frozen for further processing. The process can be broken up as follows:

Days 1 and 2: Quantification of immunoprecipitated lysate protein content; samples can be frozen until the next step.
Day 3: SDS‐PAGE, protein quantification, reduction and alkylation.
Days 3 and 4: In‐gel protease digestion.
Day 4: Peptide extraction; samples can be frozen until next step.
Day 5: Peptide desalting; samples can be frozen until next step.
Days 6 and 7: LC‐MS/MS; processing time will depend on the HPLC method and number of samples.
Days 8 and 9: Data analysis.

### Author Contributions

**Ben F. Brian 4th**: Funding acquisition; investigation; methodology; validation; visualization; writing‐original draft; writing‐review & editing. **Candace R. Guerrero**: Methodology; resources; software; validation; visualization; writing‐original draft; writing‐review & editing. **Tanya S. Freedman**: Conceptualization; data curation; formal analysis; funding acquisition; investigation; methodology; project administration; resources; software; supervision; validation; visualization; writing‐original draft; writing‐review & editing.
